# Leukocyte activation patterns in children with *Mycoplasma pneumoniae* infection: a comparison with viral and bacterial infections

**DOI:** 10.1128/spectrum.01095-25

**Published:** 2025-10-29

**Authors:** Minjing Mao, Jiacheng Zhu, Lilan Jin, Cen Jiang, Jiaoyue Mou, Xiaomeng Nie, Gang Cai

**Affiliations:** 1 Department of Laboratory Medicine, Ruijin Hospital, Shanghai Jiaotong University Medical School, Shanghai, People's Republic of China; 2 Department of Respiratory Diseases, Changzheng Hospital, the Second Military Medical University 56652 , Shanghai, People's Republic of China; City of Hope Department of Pathology, Duarte, California, USA

**Keywords:** *Mycoplasma pneumoniae*, CD64, CD169, neutrophil, monocyte

## Abstract

Respiratory infections are a leading cause of pediatric emergency department (ED) visits, necessitating accurate pathogen identification. Flow cytometry markers, CD64 (nCD64) on neutrophils and CD169 (mCD169) on monocytes, are valuable in distinguishing bacterial from viral infections. However, their roles in identifying *Mycoplasma pneumoniae* (MP), an atypical bacterium, remain unclear. This prospective study analyzed 118 children with respiratory infections and fever, categorized into MP (*n* = 64), bacterial (*n* = 20), and viral (*n* = 33) groups, alongside 28 healthy controls. Viral infections showed elevated mCD169, while bacterial infections had increased nCD64. Notably, MP infections displayed significantly higher nCD64 and mCD169 levels compared to viral and bacterial groups. These markers effectively differentiated MP infections from other pathogens. MP-infected patients were further divided into DNA^+^IgM^−^, DNA^+^IgM^+^, and DNA^−^IgM^+^ subgroups. The DNA^−^IgM^+^ group exhibited significantly lower nCD64 and mCD169 levels than the DNA^+^IgM^+^ group, with mCD169 also lower compared to the DNA^+^IgM^−^ group. These findings suggest dynamic marker changes during disease progression. In conclusion, leukocyte activation patterns, specifically nCD64 and mCD169, hold potential as diagnostic biomarkers for MP infections, aiding in distinguishing MP from other infections and improving triage and management in pediatric EDs.

**IMPORTANCE:**

Respiratory infections are one of the most frequent reasons children come to the emergency department, yet it is often difficult to quickly determine which pathogen is responsible. *Mycoplasma pneumoniae*, an atypical bacterium, is especially challenging to distinguish from common viral or bacterial infections, leading to delayed or inappropriate treatment. This study highlights the value of two immune cell markers, CD64 and CD169, measured by flow cytometry, in identifying infection patterns. We found that these markers not only distinguish bacterial from viral infections but also reveal a distinct signature in *Mycoplasma pneumoniae* cases. Moreover, marker levels appear to shift during different stages of illness, providing insights into disease progression. These findings suggest that CD64 and CD169 could become practical biomarkers to guide early diagnosis, treatment decisions, and patient triage in pediatric emergency settings, ultimately improving care for children with respiratory infections.

## INTRODUCTION

In China, *Mycoplasma pneumoniae* (MP) is the leading cause of community-acquired pneumonia cases in children over 5 years of age (1). *Mycoplasma pneumoniae* pneumonia lacks specific clinical features to distinguish it from pneumonia caused by viral or other bacterial infections (2). Delayed or inaccurate diagnosis can lead to inappropriate antibiotic use, potentially resulting in complications, such as allergic reactions, secondary infections, and an increased prevalence of antibiotic-resistant bacteria (3). Additionally, misdiagnosis may prolong the course of illness and increase healthcare costs (4). Currently, diagnostic methods for MP infections, such as polymerase chain reaction (PCR), antibody detection, and rapid antigen detection, are limited by significant drawbacks (5, 6). Thus, there is an urgent need for novel approaches that enable prompt and accurate differentiation of the underlying infection.

Research has identified several leukocyte activation markers that hold promise for diagnosing and distinguishing bacterial and viral infections: particularly the expression of CD64 (FcgRI) on neutrophils (nCD64) and CD169 (Siglec-1) on classical monocytes (mCD169) (7–11). CD64 is continuously expressed at very low densities on the surface of neutrophils in healthy individuals (7). However, its density rapidly increases in response to bacterial stimulation and their soluble products, making it a highly sensitive and specific marker for severe bacterial infections and sepsis (7, 10). Conversely, CD169 is nearly undetectable on resting monocytes but becomes significantly upregulated during viral infection (9). As such, monocyte CD169 expression (mCD169) serves as a reliable indicator of viral infection (8, 9). HLA-DR, an antigen-presenting molecule, is another critical marker; its expression on monocytes (moHLA-DR) is closely associated with their antigen-presenting function (11). Persistent immune overstimulation or exhaustion leads to reduced HLA-DR expression, which is recognized as a biomarker for poor prognosis in severe infections and sepsis (12). Flow cytometry analysis of nCD64, mCD169, and moHLA-DR has the potential to enhance diagnostic accuracy and support management of patients presenting with fever or respiratory symptoms in emergency settings.

MP is a microorganism that exhibits characteristics intermediate between viruses and common bacteria (such as gram-positive and gram-negative bacteria) (13). It lacks a cell wall and is the smallest living organism that can survive alone in nature (14). The effects of MP infections on nCD64, mCD169, and HLA-DR expression remain largely unexplored, and there is limited evidence regarding the utility of these markers for the rapid differentiation of MP infections from other types of infections. This study aimed to characterize the leukocyte activation patterns in children with MP infection and compare them to those with viral and bacterial infections.

## MATERIALS AND METHODS

### Participants and inclusion criteria

**Fig 1:**
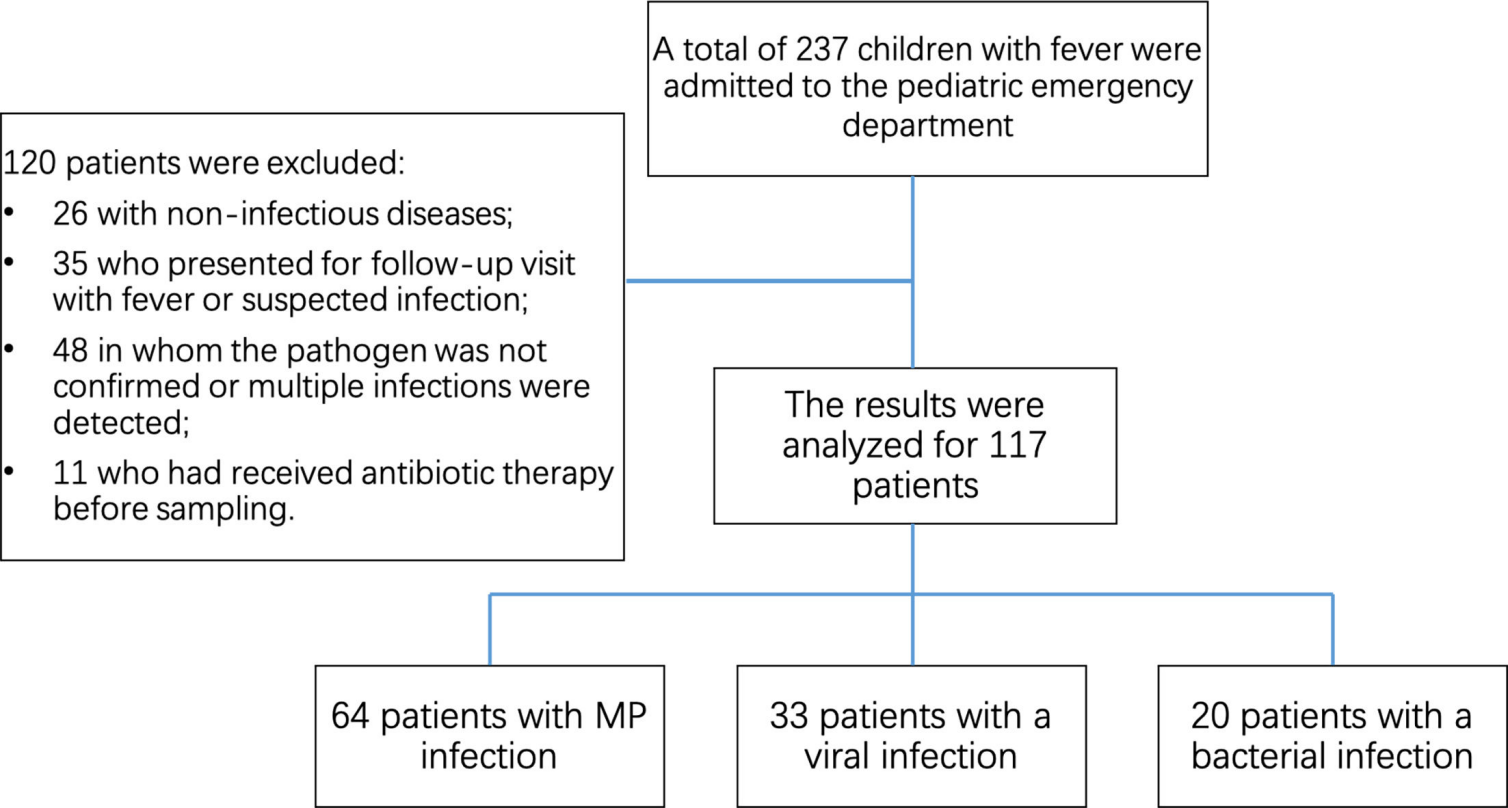
Overview of the study workflow. Illustration of the study’s sequential steps, conducted between the Emergency Department and the Clinical Laboratory of Ruijin Hospital, including the final numbers of included and excluded participants.

The recruitment population consists of patients (≤15 years of age) who presented to the pediatric emergency department at Ruijin Hospital with suspected infectious pneumonia between September 2023 and September 2024. The inclusion criteria for patients diagnosed with MP pneumonia were as follows (15): (i) clinical diagnosis of pneumonia: fever, cough, expectoration, and tachypnea (defined as a respiratory rate of ≥60 breaths/min in children <2 months, ≥50 breaths/min in 2–12 months, ≥40 breaths/min in 12–60 months, and ≥30 breaths/min in >60 months), accompanied by lung rales or coarse phlegm sounds, diminished breath sounds, and pulmonary infiltrates on chest imaging; (ii) evidence of MP infection: serum MP antibody titer ≥ 1:80 or a positive nasopharyngeal polymerase chain reaction result. The inclusion criteria for patients with non-MP pneumonia were as follows: (i) symptoms of respiratory infection with a clinical diagnosis of pneumonia not attributable to MP; (ii) confirmed infection with other pathogens, such as viruses and *Streptococcus pneumoniae*; and (iii) serum MP antibody titer ≤ 1:80 during hospitalization. Exclusion criteria included unknown infection status, multiple infections (viral-bacterial, viral-MP, or MP-bacterial coinfection), chronic viral infectious diseases, granulocyte colony-stimulating factor therapy, myelodysplastic syndrome, transplantation, autoimmune diseases, and autoinflammatory diseases, as these conditions are associated with reported upregulation of mCD169 and nCD64 (16, 17). Patients who presented for follow-up visits due to fever or suspected infection, typically more than 7 days after symptom onset, were also excluded from the study cohort (Fig. 1). Blood samples from the healthy control group were obtained from children undergoing routine physical examinations at our hospital’s maternal and child health center.

A total of 64 patients were enrolled in the MP infection group. For comparison, 20 patients with other bacterial infections (bacterial infection group) and 33 patients with viral infections (viral infection group) were enrolled. Among the 17 common pathogens identified in non-MP patients (Table S1), the most frequent were *Streptococcus pneumoniae* (40.0%) in the bacterial infection group and *influenza A/B* virus (42.8%) in the viral infection group. The clinical and biological characteristics of all participants are summarized in Table 1.

**TABLE 1:** Characteristics of the study cohort^*a*^

	Healthy control (*n* = 28)	MP infection (*n* = 64)	Viral infection (*n* = 33)	Bacterial infection (*n* = 20)	*P* value
Time between the first symptoms and sampling (day)					
Median (25% quantile, 75% quantile)		3 (2, 5)	4 (1, 6)	3 (2, 5)	0.725
Age					
Median (25% quantile, 75% quantile)	6.00 (5.00, 8.00)	8.00 (6.00, 9.00)	8.20 (6.00, 10.3)	6.50 (5.00, 8.25)	0.211
<2 years (%)	0 (0.00%)	1 (1.57%)	1 (3.00%)	0 (0.00%)	0.127
2–6 years (%)	15 (53.6%)	17 (26.6%)	10 (30.3%)	10 (50.0%)	
>6 years (%)	13 (46.4%)	48 (75.0%)	22 (66.7%)	10 (50.0%)	
Gender					
Male (%)	13 (46.4%)	27 (42.2%)	15 (45.5%)	11 (55.0%)	0.796
Female (%)	15 (53.6%)	37 (57.8%)	18 (54.5%)	9 (45.0%)	
Clinical symptom					
Fever (%)		53 (82.8%)	26 (78.8%)	19 (95.0%)	0.287
Cough (%)		62 (96.9%)	33 (100%)	20 (100%)	0.431
Chills (%)		13 (20.3%)	6 (18.2%)	5 (25.0%)	0.359
Rhinorrhea (%)		39 (60.9%)	20 (60.6%)	10 (50.0%)	0.804
Dyspnea (%)		9 (14.0%)	6 (18.2%)	2 (10.0%)	0.706
Vomiting (%)		1 (1.6%)	1 (3.0%)	0 (0.0%)	0.705
Diarrhea (%)		1 (1.6%)	1 (3.0%)	2 (10.0%)	0.191
Sore throat (%)		5 (7.8%)	4 (12.1%)	2 (10.0%)	0.969
Loss of appetite (%)		32 (50%)	17 (51.5%)	12 (60.0%)	0.734
Biological index: median (25% quantile, 75% quantile)					
Leukocytes (×10^9^/L)		6.47 (5.44, 7.86)^*c*^	6.14 (5.46, 7.84)^*c*^	11.2 (7.36, 13.4)	<0.001
Neutrophil frequency (%)		60.0 (48.5, 69.3)^*b*^^,*c*^	74.8 (62.3, 80.6)	74.6 (70.0, 77.3)	<0.001
Lymphocyte frequency (%)		32.2 (22.0, 41.4)^*b*^^,*c*^	13.7 (10.4, 22.8)	18.0 (14.1, 22.2)	<0.001
Monocyte frequency (%)		7.00 (5.27, 8.80)^*b*^	9.22 (6.70, 12.1)^*c*^	6.30 (5.18, 8.35)	0.005
Neutrophil (×10^9^/L)		3.95 (2.76, 4.83)^*c*^	4.62 (3.41, 5.74)^*c*^	9.00 (5.22, 10.1)	<0.001
Lymphocytes (×10^9^/L)		1.96 (1.47, 2.80)^*b*^	0.92 (0.64, 1.49)^*c*^	1.76 (1.55, 2.16)	<0.001
Monocytes (×10^9^/L)		0.46 (0.34, 0.58)^*b*^^,*c*^	0.61 (0.44, 0.66)	0.66 (0.49, 0.90)	<0.001
B cells (/μL)		362 (252, 535)^*b*^	173 (116, 270)^*c*^	402 (225, 574)	<0.001
T cells (/μL)		1,384 (947, 1,952)^*b*^	731 (467, 975)^*c*^	1,160 (1,050, 1,491)	<0.001
Th cells (/μL)		801 (505, 1,064)^*b*^	347 (259, 544)^*c*^	613 (552, 879)	<0.001
Tc cells (/μL)		462 (320, 682)^*b*^	254 (176, 391)^*c*^	443 (376, 637)	<0.001
NK cells (/μL)		148 (84.6, 249)^*b*^	62.5 (42.9, 165)	110 (84.4, 196)	0.012
C-reactive protein (mg/L)		8.00 (4.82, 16.1)	6.05 (2.09, 11.7)^*c*^	14.2 (7.04, 23.2)	<0.001
HBP (ng/mL)		36.6 (21.4, 58.0)^*b*^^,*c*^	21.1 (11.0, 38.2)^*c*^	110 (90.5, 152)	<0.001
IFN-α (pg/L)		6.80 (3.60, 16.1)	8.95 (4.47, 49.8)	5.20 (2.40, 12.5)	0.104
IFN-γ (pg/L)		17.1 (4.73, 87.4)	18.9 (6.22, 129)	16.6 (3.80, 52.0)	0.817
IL-6 (pg/mL)		8.70 (3.55, 21.2)	19.5 (6.35, 34.5)	19.6 (5.55, 37.7)	0.093
IL-8 (pg/mL)		3.90 (2.40, 13.2)	12.2 (3.05, 23.7)	22.4 (4.10, 33.9)	0.025
IL-10 (pg/mL)		2.50 (2.40, 3.32)^*b*^	6.31 (3.45, 15.2)	3.80 (2.40, 5.85)	<0.001

^*a*^ For normally distributed quantitative data, the mean ± standard deviation was used and one-way ANOVA was employed to calculate the *P* value, followed by the Bonferroni test for pairwise comparisons. For non-normally distributed quantitative data, the median (interquartile range) was used, and the nonparametric Kruskal-Wallis test was applied to calculate the *P* value, followed by Dunn’s test for pairwise comparisons. Categorical variables are presented as frequencies, and the Chi-square test was used to calculate *P* values.

^*b*^ *P* < 0.05 vs viral infection group.

^*c*^ *P* < 0.05 vs bacterial infection group.

### Microbiological and biochemical analysis

Samples collected from suspected infection sites (including blood, bronchial secretions, bronchoalveolar lavage fluid, nasopharyngeal swabs, etc.) were analyzed using direct detection methods to identify bacteria, fungi, and/or viruses. The diagnostic approaches included routine culture, nucleic acid amplification testing or metagenomics analysis, and serological or antigen testing, all conducted in accordance with international and local guidelines.

The serum heparin-binding protein (HBP) levels were measured using an Axis-Shield clinical chemistry HBP assay (Axis-Shield Diagnostics Ltd., Dundee, Scotland). C-reactive protein (CRP) levels were determined using a polystyrene-enhanced immunonephelometric method on the Dimension Vista System (Siemens). Peripheral blood leukocyte counts and lymphocyte subset analyses were performed using a Sysmex analyzer and BD Canto II Flow Cytometry, following the manufacturer’s instructions. Serum cytokine levels, including IFN-α, IFN-γ, IL-6, IL-8, and IL-10, were quantified using a Cytometric Bead Array. All assays were conducted exclusively for research purposes and were systematically applied to all enrolled patients.

### Leukocyte activation profile by flow cytometry

**Fig 2:**
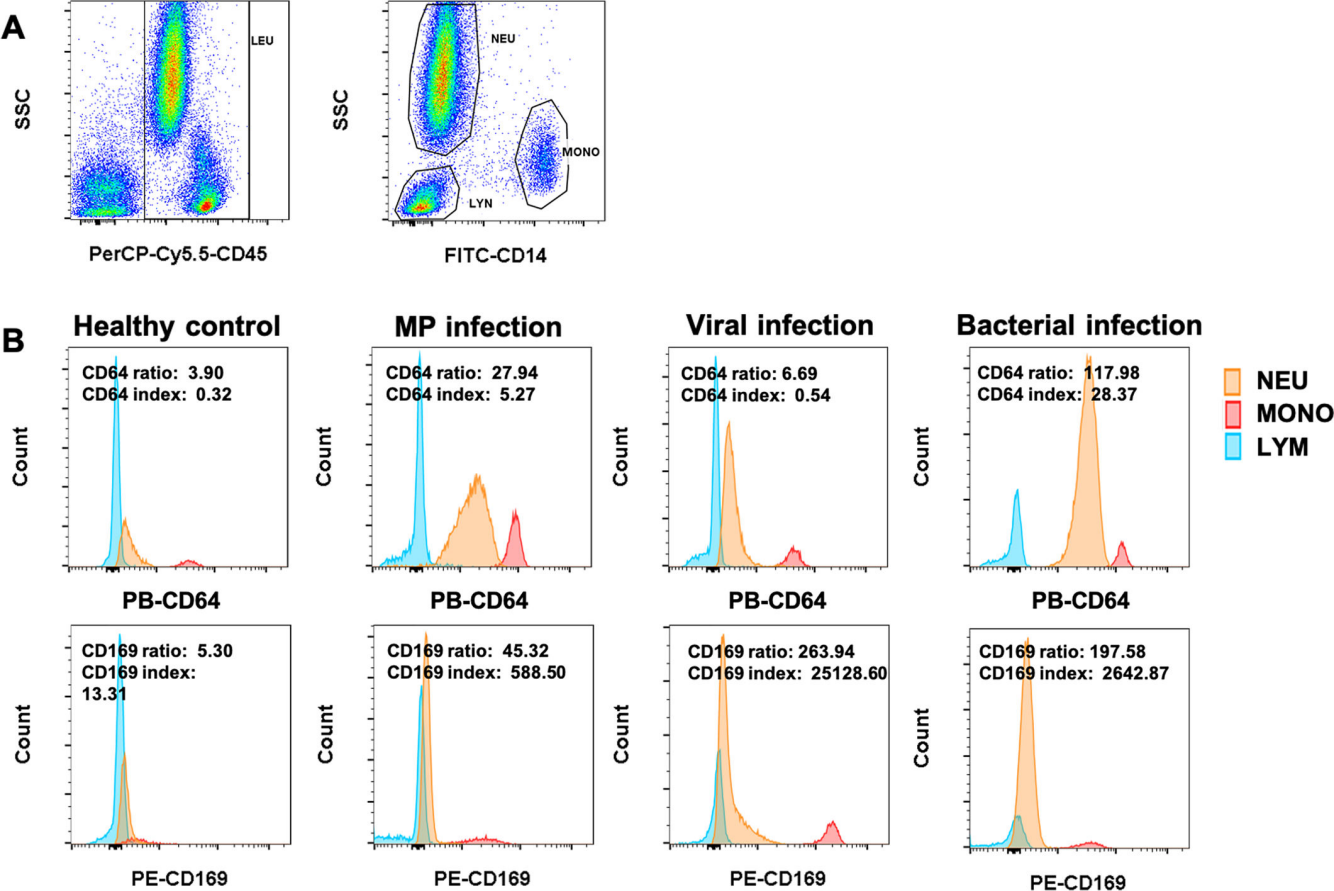
Gating strategy. (**A**) Leukocytes were gated based on SSC vs CD45. Monocytes, neutrophils, and lymphocytes are subsequently identified as intermediate SSC/CD14+, high SSC/CD14−, and low SSC/CD14−, respectively. (**B**) Representative histograms of CD169 and CD64 expression on lymphocytes, neutrophils, and monocytes for healthy controls, MP-infected patients, and those with viral or bacterial infections. Marker ratios and indexes were calculated as described in Materials and Methods. MFI, median fluorescence intensity.

Leukocyte activation was assessed by measuring CD169, CD64, and HLA-DR markers on monocytes, neutrophils, and lymphocytes using flow cytometry. Ethylenediaminetetraacetic acid (EDTA)-anticoagulated blood samples were processed using a one-step procedure. Briefly, 50 µL of EDTA whole blood was mixed with 10 µL of the following antibodies: anti-CD169-PE (R-Phycoerythrin, clones 7-239), anti-CD64-PB (Pacific Blue, clone 22), anti-HLA-DR-APC (Allophycocyanin, clone Immu357), anti-CD45-PercP-Cy5.5 (peridinin chlorophyll protein-Cyanine5.5, clone 2D1), and anti-CD14-FITC (fluorescein isothiocyanate, clone M5E2). Samples were incubated at room temperature in the dark for 20 minutes, followed by erythrocyte lysis with 450 µL of hemolysin. After complete lysis, flow cytometric analysis was performed using a BD Canto II flow cytometer equipped with three lasers and eight detection channels (BD, USA). Data were acquired for 100,000 leukocyte events. For data analysis, leukocytes were gated using side scatter (SSC) vs CD45, and populations of neutrophils, lymphocytes, and monocytes were then identified based on an SSC-CD14 dot plot as follows: SSC^high^/CD14^neg^ (neutrophils), SSC^low^/CD14^neg^ (lymphocytes), and SSC^intermediate^/CD14^pos^ (monocytes) (Fig. 2A).

The median fluorescence intensity (MFI) was measured in the phycoerythrin (CD169), Pacific Blue (CD64), and Allophycocyanin (HLA-DR) channels for each cell population.

Neutrophil CD64 expression levels (nCD64): quantified as the CD64 ratio and CD64 index.

CD64 ratio: ratio of CD64 MFI on neutrophils (NEU) to CD64 MFI on lymphocytes (LYM).

CD64 index: calculated as nCD64 index = (MFI_NEU CD64_/MFI_LYM CD64_)/(MFI_MON CD64_/MFI_NEU CD64_), using the MFI of CD64 on lymphocytes and monocytes as internal negative and positive controls.

Monocyte CD169 expression levels (mCD169): quantified as the CD169 ratio and CD169 index.

CD169 ratio: ratio of CD169 MFI on monocytes to CD169 MFI on lymphocytes (LYM).

CD169 index: calculated as CD169 index = (MFI_MON CD169_/MFI_LYM CD169_)/(MFI_NEU CD169_/MFI_MON CD169_), using the MFI of CD169 on lymphocytes and neutrophils as internal negative and positive controls.

Since no pure HLA-DR-negative internal reference population was available, moHLA-DR expression levels were calculated using the geometric mean MFI. Daily calibration with CS&T microbeads ensured consistent and robust MFI measurements throughout the study.

### Statistical analysis

Statistical analyses were conducted using GraphPad Prism version 10.1.2. Qualitative variables were expressed as frequencies and percentages and compared between groups using the Chi-square or Fisher’s exact test, as appropriate. Quantitative variables were presented as either mean ± standard error or median with interquartile range, depending on their distribution. For data not following a normal distribution, the Kruskal-Wallis test was used, followed by Dunn’s multiple comparison test. For normally distributed data, a one-way ANOVA test was performed, with the Holm-Šídák method applied for *post hoc* multiple comparisons. Correlation between variables was assessed with the Spearman correlation coefficient.

The receiver operating characteristic (ROC) curve was generated to illustrate the trade-off between true positive rates (sensitivity) and false-positive rates (1 − specificity) across all possible cutoff values. The area under the ROC curve (AUC) was calculated to quantify the diagnostic performance. A two-tailed *P* value of less than 0.05 was considered statistically significant for all tests.

## RESULTS

### Clinical characteristics of patients

During the initial study period, 238 patients admitted to the Emergency Department at Ruijin Hospital were screened. Blood samples were collected on the day of admission and analyzed using flow cytometry. Of these, only 118 patients met the inclusion and exclusion criteria. These patients were divided into three groups: the MP infection group (*n* = 64), the viral infection group (*n* = 33), and the bacterial infection group (*n* = 20). Additionally, 28 healthy controls were included. An overview of the study workflow is provided in Fig. 1, and the clinical characteristics of the enrolled patients are summarized in Table 1. No significant differences in age, gender, or sampling time post-onset of symptoms were observed among the four groups.

Electronic medical records were reviewed for each patient. During the acute infection phase, the most common symptoms across all groups were cough (92.9%), fever (86.7%), rhinorrhea (61.1%), loss of appetite (53.9%), and chills (21.2%). Less frequently reported symptoms included dyspnea (15.0%), sore throat (9.7%), diarrhea (3.5%), and vomiting (1.8%). However, no significant differences in these clinical symptoms were observed among the three patient groups (Table 1).

Biochemical and biological measurements revealed significant differences among the MP, viral, and bacterial groups in peripheral leukocyte counts and differentials, lymphocyte subset counts, CRP, and HBP levels. Cytokine levels of IFN-α, IL-8, and IL-10 differed significantly among the groups, while no significant differences were observed for IFN-γ and IL-6 (Table 1).

### Leukocyte activation patterns in pediatric infections

We analyzed the expression of CD64, CD169, and HLA-DR markers on leukocytes from the included children using the gating strategy depicted in Fig. 2A. Neutrophil CD64 expression levels were represented by the CD64 ratio and CD64 index, while monocyte CD169 expression levels were captured using the CD169 ratio and CD169 index. Representative data for each group are shown in Fig. 2B.

**Fig 3:**
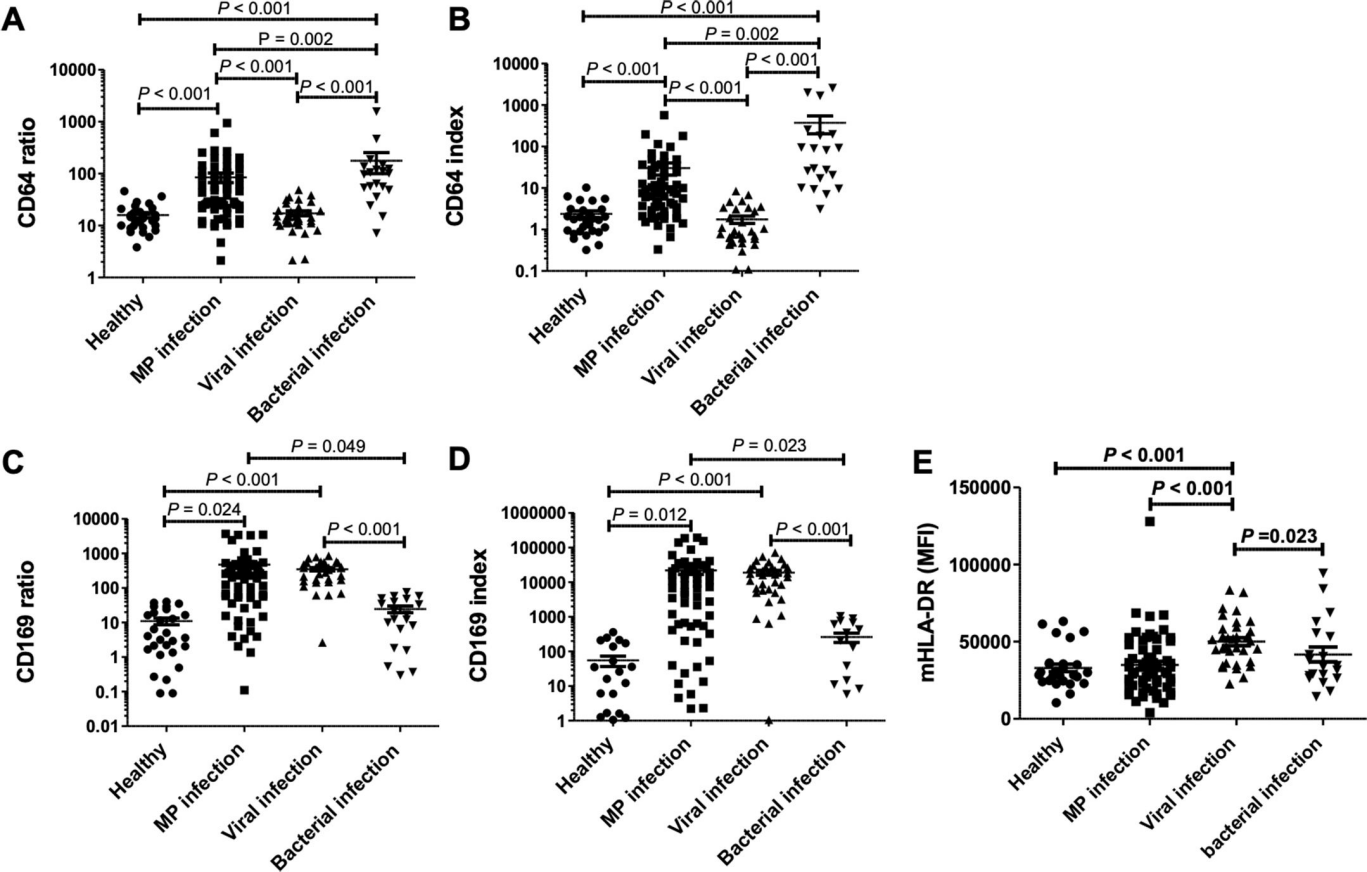
Biomarker levels in the four groups of children. The levels of (**A**) CD64 ratio, (**B**) CD64 index, (**C**) CD169 ratio, (**D**) CD169 index, and (**E**) MFI of HLA-DR on monocytes were compared among healthy controls, children with MP infections, viral infections, and bacterial infections. Statistical analysis was performed using the Kruskal-Wallis test, followed by Dunn’s multiple comparison test, with significant *P*-values indicated.

As shown in Fig. 3, nCD64 levels were significantly elevated in bacterial infection patients compared to healthy controls (CD64 ratio: *P* = 0.001; CD64 index: *P* < 0.001) and viral infection patients (CD64 ratio: *P* < 0.001; CD64 index: *P* < 0.001) (Fig. 3A and B). Similarly, mCD169 expression was notably higher in viral infection patients compared to both healthy controls (CD169 ratio: *P* = 0.023; CD169 index: *P* = 0.014) and bacterial infection patients (CD169 ratio: *P* = 0.042; CD169 index: *P* = 0.021) (Fig. 3C and D). Children with MP infection exhibited augmented mCD169 expression and nCD64 (Fig. 3A through D). Children with MP infection exhibited comparable levels of mCD169 to those with viral infection (CD169 ratio: *P* = 0.384; CD169 index: *P* = 0.695), which were significantly higher than those in the healthy control (CD169 ratio: *P* < 0.001; CD169 index: *P* < 0.001) and bacterial infection groups (CD169 ratio: *P* < 0.001; CD169 index: *P* < 0.001). Although nCD64 levels in MP patients were lower than in bacterial infection patients (CD64 ratio: *P* = 0.002; CD64 index: *P* = 0.002), they were markedly higher than in healthy control (CD64 ratio: *P* < 0.001; CD64 index: *P* < 0.001) and viral infection patients (CD64 ratio: *P* < 0.001; CD64 index: *P* < 0.001).

**Fig 4:**
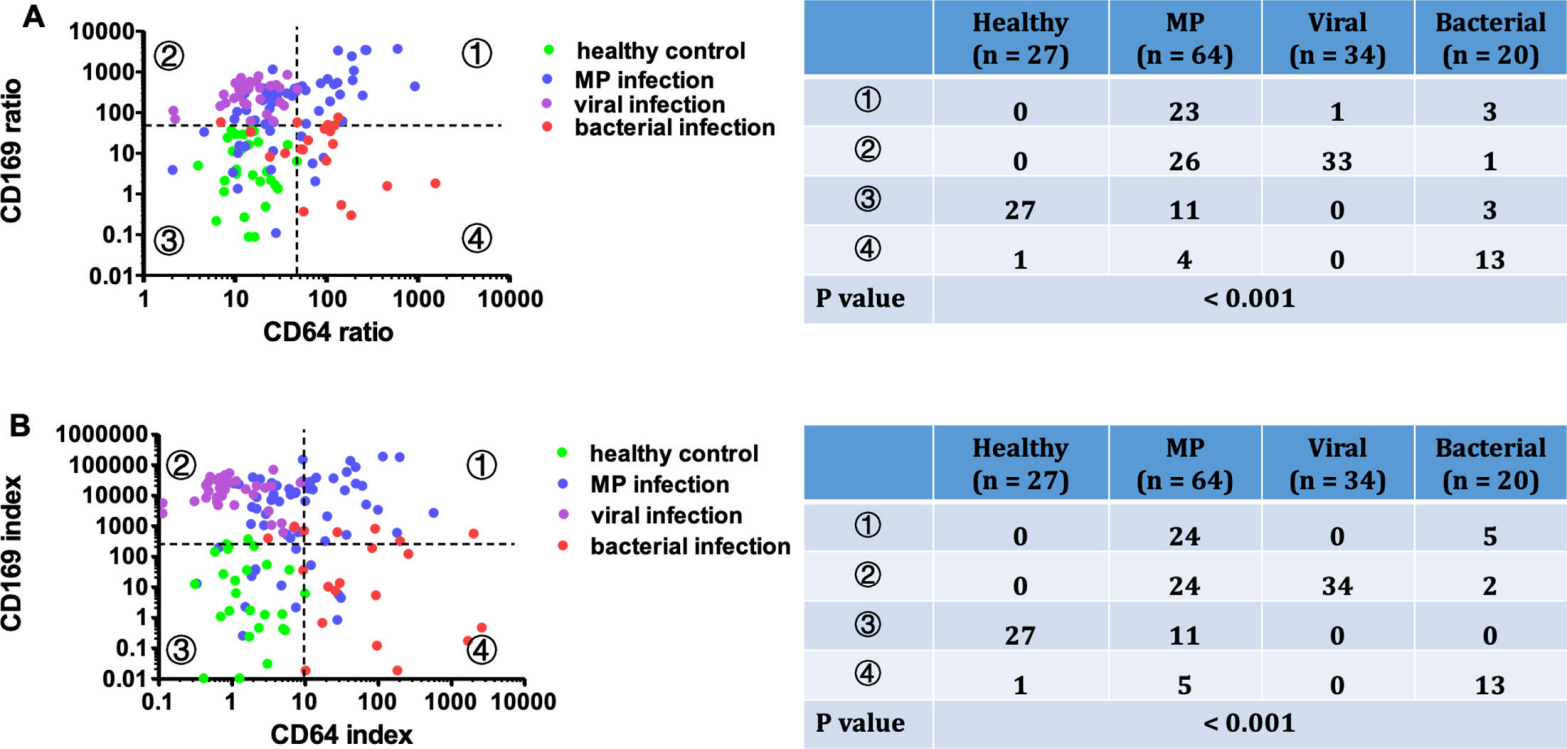
Relationship between nCD64 and mCD169 across four groups. Scatterplots showing the distribution of subjects in (**A**) CD64 ratio/CD169 ratio scatterplot and (**B**) CD64 index/CD169 index plots. Quadrants were defined using the mean + 3 SD of the healthy control population as cutoff values. The number of cases per group within each quadrant and the chi-squared test results are presented alongside the plots.

To further explore leukocyte activation patterns, we plotted nCD64 and mCD169 on a dual-coordinate system, dividing the plot into four quadrants using cutoff values derived from the mean plus three standard deviations of the healthy control group (CD64 ratio: 44.43; CD169 ratio: 49.58; CD64 index: 9.06; CD169 index: 346.65). The distribution of cases varied distinctly among the groups. As shown in Fig. 4, nearly all healthy controls (27/28, 96.4%) clustered in the third quadrant on both the CD64 ratio/CD169 ratio and CD64 index/CD169 index plots. In contrast, most viral infection cases were confined to the second quadrant, with 97.0% (32/33) and 100% (33/33) falling within this region on the respective plots. Bacterial infection cases predominantly occupied the fourth quadrant, with 65.0% (13/20) located in this area across both plots. The distribution for MP-infected patients was more dispersed, with cases spread across all quadrants. Specifically, on the CD64 ratio/mCD169 ratio plot, MP cases were distributed as follows: 35.9% (23/64) in the first quadrant, 40.6% (26/64) in the second, 17.2% (11/64) in the third, and 6.3% (4/64) in the fourth. Similarly, the CD64 index/mCD169 index plot showed 37.5% (24/64) of MP cases in both the first and second quadrants, 17.2% (11/64) in the third, and 7.8% (5/64) in the fourth. Statistical analysis confirmed significant differences in the distribution patterns among the four groups across both plots (*P* < 0.001).

Finally, we assessed HLA-DR expression on monocytes, a marker of immune activation (11, 12). HLA-DR levels varied significantly among groups (healthy vs MP vs viral vs bacterial: 32,993 ± 2,493 vs 35,210 ± 2,128 vs 50,768 ± 2,608 vs 41,739 ± 7,859; *P* < 0.001). Viral infection patients exhibited higher HLA-DR levels, significantly surpassing those in healthy controls (*P* < 0.001) and MP-infected patients (*P* < 0.001, Fig. 3E).

### Diagnostic value of nCD64 and mCD169 for MP infection

**Fig 5:**
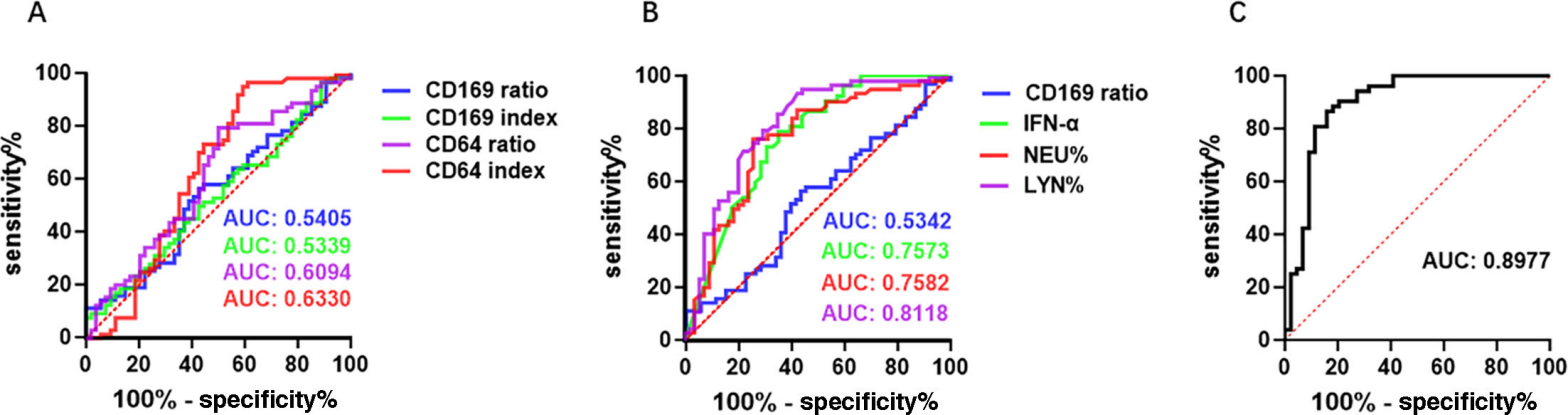
ROC analysis for biomarker performance. (**A**) ROC curves for distinguishing MP infections from viral and bacterial infections using the CD169 ratio, CD169 index, CD64 ratio, and CD64 index. (**B**) ROC curves for differentiating MP infections from both viral and bacterial infections using individual markers, including the CD169 ratio, interferon (IFN)-α, neutrophil frequency (NEU%), and lymphocyte frequency (LYN%). (**C**) ROC curves for differentiating MP infections from both viral and bacterial infections using a combination of the markers from panel B. The area under the curve is provided for each ROC analysis.

Consistent with findings from previous studies (10, 18–22), our results confirmed that nCD64 and mCD169 are highly effective in distinguishing bacterial from viral infections (data not shown). Building on this, our primary focus was to determine whether these biomarkers could effectively differentiate MP infections from other etiologies. ROC curve analysis was conducted to evaluate the diagnostic performance of these markers in distinguishing MP infection from healthy controls, as well as from viral and bacterial infection. Compared to healthy controls, both mCD169 and nCD64 demonstrated strong diagnostic utility for MP infection in children. The AUC values for CD64 ratio, CD64 index, CD169 ratio, and CD169 index were 0.8195, 0.8412, 0.8984, and 0.8956, respectively (Fig. S1A). Binary logistic regression further enhanced the diagnostic accuracy, with a combination of CD169 ratio, CD64 index, and CD64 ratio achieving a maximum AUC of 0.9492 (Fig. S1B). In distinguishing MP infections from viral infections, the AUC values for CD64 ratio and CD64 index were 0.7827 and 0.8857, respectively (Fig. S1C). Incorporating the CD64 ratio together with CD3^+^ T-cell and monocyte counts further improved the diagnostic performance, yielding a maximum AUC of 0.9268 (Fig. S2A). Similarly, when comparing MP with bacterial infections, the AUC values were 0.8391 for the CD64 ratio and 0.8297 for the CD64 index. Adding the CD64 index, HBP, and lymphocyte percent (LYN%) into the model increased the AUC to 0.9031 (Fig. S2B). When all patients with viral and bacterial infections were combined as the control group, nCD64 rather than mCD169 remained an effective marker for identifying MP infection (Fig. 5A). The AUC values for the CD64 ratio and CD64 index were 0.6094 (*P* = 0.035) and 0.6330 (*P* = 0.027), respectively, with optimal cutoff values of 19.98 (sensitivity = 79.69% and specificity = 50.00%) and 1.175 (sensitivity = 95.32% and specificity = 59.26%). By contrast, the AUC values for the CD169 ratio and CD169 index were 0.5405 (*P* = 0.125) and 0.5339 (*P* = 0.325), respectively. However, combining the CD169 ratio with IFN-α, NEU%, and LYN% demonstrated robust diagnostic performance, achieving an AUC value of 0.8977 (Fig. 5B and C).

Further analysis of the data from MP-infected children revealed a significant positive correlation between nCD64 levels and various inflammatory markers. Specifically, nCD64 showed strong correlations with CRP (CD64 ratio: *r* = 0.589, *P* < 0.001; CD64 index: *r* = 0.658, *P* < 0.001), IFN-γ (CD64 ratio: *r* = 0.412, *P* = 0.002; CD64 index: *r* = 0.366, *P* = 0.007), IL-6 (CD64 ratio: *r* = 0.521, *P* = 0.000; CD64 index: *r* = 0.529, *P* < 0.001), and IL-8 (CD64 ratio: *r* = 0.536, *P* < 0.001; CD64 index: *r* = 0.535, *P* < 0.001). Additionally, significant correlations were observed between nCD64 levels and lymphocyte subset composition. MP-infected children exhibited an elevated proportion of B cells (CD64 ratio: *r* = 0.472, *P* < 0.001; CD64 index: *r* = 0.438, *P* < 0.001) and reduced proportion of total T cells (CD64 ratio: *r* = −0.304, *P* = 0.014; CD64 index: *r* = −0.280, *P* = 0.025) and cytotoxic T-cells (Tc cells) (CD64 ratio: *r* = −0.268, *P* = 0.032; CD64 index: *r* = −0.274, *P* = 0.028). Regarding mCD169, its index level demonstrated significant positive correlations with IL-10 (*r* = 0.372, *P* = 0.007), IFN-α (*r* = 0.536, *P* < 0.001), and IFN-γ (*r* = 0.470, *P* < 0.001), alongside negative correlations with total T cell counts (*r* = −0.258, *P* = 0.040) and Tc-cell counts (*r* = −0.264, *P* = 0.035). The CD169 ratio, however, showed no significant correlation with most laboratory indices, except for positive correlations with the proportion of B cells (*r* = 0.286, *P* = 0.022), and cytokines IFN-α (*r* = 0.535, *P* < 0.001) and IFN-γ (*r* = 0.560, *P* < 0.001) (Table S2).

### Correlation of leukocyte activation markers with MP PCR/IgM test patterns

**Fig 6:**
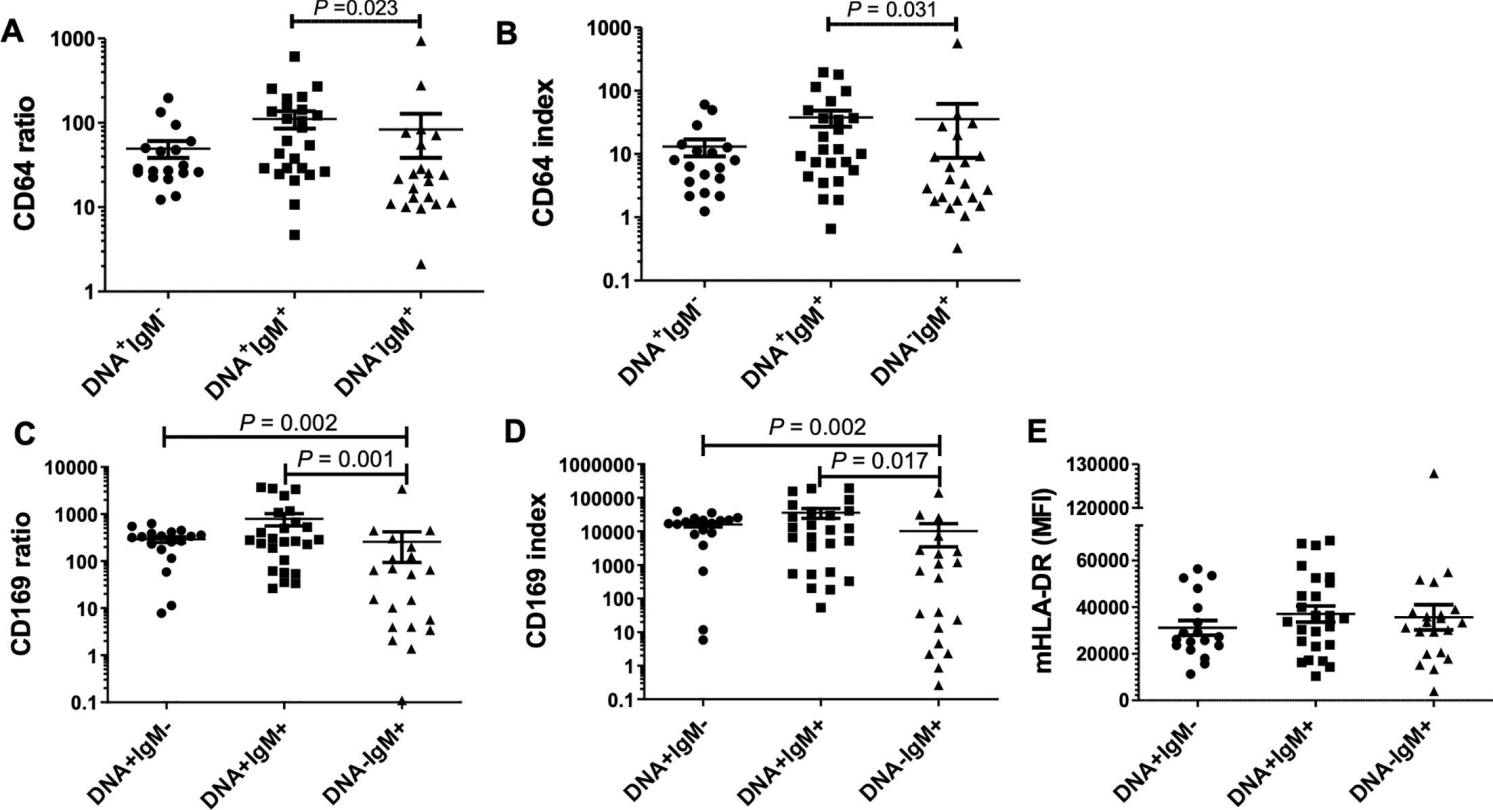
Comparison of biomarker levels across the DNA^+^IgM^-^, DNA^+^IgM^+^, and DNA^-^IgM^+^ groups of MP-infected children. (**A**) CD64 ratio, (**B**) CD64 index, (**C**) CD169 ratio, (**D**) CD169 index, and (**E**) MFI of HLA-DR on monocytes were compared among three groups. Statistical analysis was conducted using the Kruskal-Wallis test, followed by Dunn’s multiple comparison test, with significant *P*-values indicated.

To investigate whether leukocyte activation markers (mCD169, nCD64, and moHLA-DR) correlate with MP testing patterns, MP-infected children were categorized into three groups—DNA^+^IgM^-^, DNA^+^IgM^+^, and DNA^-^IgM^+^—based on pharyngeal swab PCR and serological test results. The analysis revealed that patients in the DNA^-^IgM^+^ group exhibited significantly lower mCD169 and nCD64 levels compared to those in the DNA^+^IgM^+^ group (CD169 ratio: *P* = 0.001; CD169 index: *P* = 0.017; CD64 ratio: *P* = 0.023; and CD64 index: *P* = 0.031) (Fig. 6). Furthermore, mCD169 levels in the DNA^-^IgM^+^ group were notably lower than those observed in the DNA^+^IgM^-^ group (CD169 ratio: *P* = 0.002; CD169 index: *P* = 0.002) (Fig. 6). Conversely, monocyte HLA-DR expression remained consistent across all three groups (Fig. 6E). Laboratory metrics also revealed differences among the groups, particularly in levels of IFN-γ (*P* < 0.001), IL-6 (*P* = 0.017), IL-10 (*P* = 0.018), neutrophil frequency (*P* = 0.010), and lymphocyte frequency (*P* = 0.009) (Table 2).

**TABLE 2:** Demographic and analytical parameters in different MP infection population^*a*^

	DNA^+^IgM^−^ (*n* = 18)	DNA^+^IgM^+^ (*n* = 25)	DNA^−^IgM^+^ (*n* = 21)	*P*-value
Age				
Mean ± SD	7.28 ± 2.19	7.56 ± 2.60	8.29 ± 2.80	0.441
Gender				
Male/female	10 (55.6%)	10 (40.0%)	7 (33.3%)	0.360
Female	8 (44.4%)	15 (60.0%)	14 (66.7%)	
Biological index				
Leukocytes (×10^9^/L)	7.14 (4.88, 7.83)	6.76 (5.49, 8.12)	6.26 (5.52, 6.61)	0.671
Neutrophil frequency (%)	62.1 ± 11.2^*b*^	63.3 ± 12.9^*b*^	51.9 ± 14.6	0.010
Lymphocyte frequency (%)	29.8 ± 9.65	28.3 ± 11.1^*b*^	38.5 ± 13.1	0.009
Monocyte frequency (%)	6.84 ± 3.19	6.99 ± 2.38	7.53 ± 2.97	0.715
Neutrophil (×10^9^/L)	4.27 (2.72, 5.22)	4.24 (3.28, 5.39)	2.93 (2.55, 3.95)	0.076
Lymphocytes (×10^9^/L)	1.79 (1.43, 2.09)	1.77 (1.16, 2.94)	2.45 (1.90, 2.80)	0.226
Monocytes (×10^9^/L)	0.40 (0.33, 0.62)	0.47 (0.36, 0.53)	0.53 (0.33, 0.58)	0.748
B cells (/μL)	387 (295, 486)	352 (254, 500)	385 (243, 606)	0.845
T cells (/μL)	1,152 (948, 1,641)	1,316 (678, 1,919)	1,807 (1,389, 2,154)	0.114
Th cells (/μL)	757 ± 408	742 ± 395	988 ± 469	0.112
Tc cells (/μL)	409 (319, 492)	396 (258, 817)	630 (439, 696)	0.118
NK cells (/μL)	148 (112, 301)	99.2 (60.9, 228)	149 (110, 215)	0.303
CRP (mg/L)	14.0 (6.25, 24.5)	9.00 (5.00, 16.0)	6.50 (3.00, 9.00)	0.056
HBP (ng/mL)	44.5 (27.9, 70.6)	42.9 (21.7, 76.7)	31.3 (16.2, 36.8)	0.196
IFN-α (pg/L)	8.55 (4.93, 16.1)	6.75 (3.53, 18.1)	4.3 (2.50, 14.1)	0.231
IFN-γ (pg/L)	43.4 (5.95, 104)	33.8 (8.58, 134)^*b*^	5.10 (3.13, 15.6)	0.026
IL-6 (pg/mL)	9.55 (3.70, 22.3)	11.7 (7.03, 29.7)^*b*^	3.95 (2.40, 9.72)	0.017
IL-8 (pg/mL)	2.75 (2.40, 7.00)	3.90 (2.40, 13.4)	4.80 (2.40, 19.6)	0.633
IL-10 (pg/mL)	2.55 (2.40, 3.57)	3.25 (2.48, 4.15)^*b*^	2.40 (2.40, 2.50)	0.018

^*a*^ For normally distributed quantitative data, the mean ± standard deviation was used, and one-way ANOVA was employed to calculate the *P* value, followed by the Bonferroni test for pairwise comparisons. For non-normally distributed quantitative data, the median (interquartile range) was used, and the nonparametric Kruskal-Wallis test was applied to calculate the *P* value, followed by Dunn’s test for pairwise comparisons. Categorical variables are presented as frequencies, and the Chi-square test was used to calculate *P* values.

^*b*^ *P* < 0.05 vs DNA^−^IgM^+^ group.

**Fig 7:**
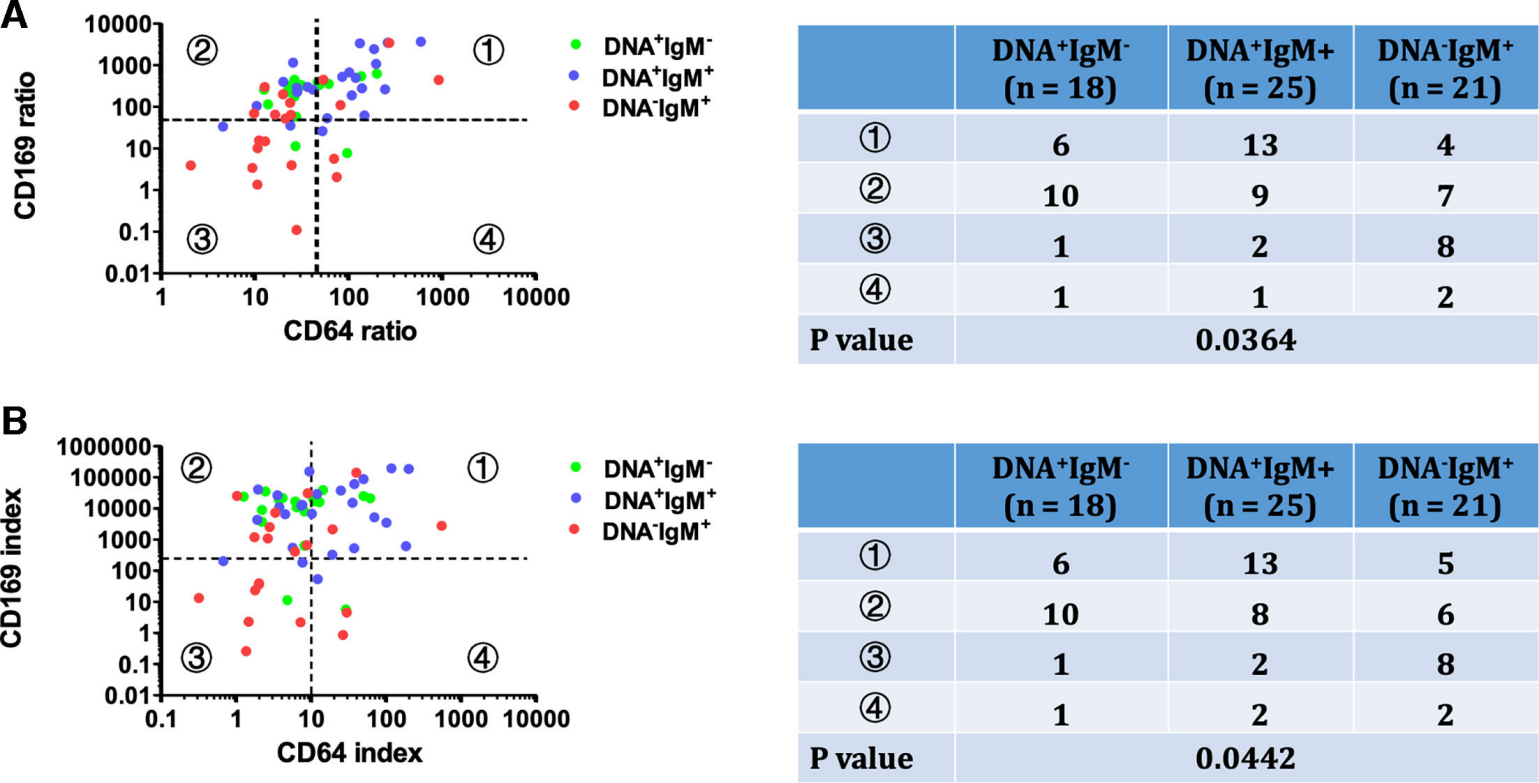
Distribution of nCD64 and mCD169 in MP-infected subgroups. MP-infected patients were categorized into three groups as described in Fig. 6. Their distribution is illustrated in (**A**) the CD64 ratio/CD169 ratio scatterplot and (**B**) the CD64 index/CD169 index scatterplot. The number of cases in each subgroup within the quadrants, along with the results of the chi-squared analysis, is presented on the right side of the scatterplots.

Using the cutoff values defined in Fig. 4, the distribution of these subgroups was analyzed on nCD64/mCD169 two-parameter plots. Significant differences were observed in the distribution patterns among the DNA^+^IgM^−^, DNA^+^IgM^+^, and DNA^−^IgM^+^ groups on both the CD64 ratio/CD169 ratio plot (*P* = 0.0364) and the CD64 index/CD169 index plot (*P* = 0.0442) (Fig. 7).

## DISCUSSION

This study demonstrates that, unlike viral or bacterial infection alone—which predominantly upregulates mCD169 and nCD64 expression, respectively (7, 9, 10, 18–23), MP infection specifically induces a synchronized phenomenon of neutrophil CD64 expression and monocyte CD169, resulting in a distinctive pattern of leukocyte activation.

The precise mechanisms underlying this leukocyte activation pattern remain unclear. One plausible explanation involves the pathogen-associated molecular patterns of MP, which may activate the MyD88-NF-κB signaling pathway in innate immune cells (24). This pathway has been implicated in the upregulation of CD64 and CD169 across various immune cell types (25, 26). Additionally, MP-mediated induction of cytokines, particularly IFN, may play a role. *In vitro* studies have demonstrated that type I IFNs (α/β/ω) robustly and rapidly induce CD169 expression, while type II IFN (γ) is known to regulate CD64 expression (17, 27). Interestingly, no significant differences in serum IFN levels were observed between MP-infected patients and those infected with viral or bacterial infections (Table 1). However, within the MP-infected cohort, positive correlations were detected between serum IFN-γ levels and both nCD64 and mCD169, as well as between IFN-α and mCD169 (Table S2).

Respiratory tract infections are a leading cause of emergency room visits in children (28). Differentiating the sources of these infections poses a significant challenge for pediatricians, as clinical symptoms often overlap and reliable biomarkers remain limited (4, 29). Given the distinct leukocyte activation patterns observed between patients with viral, other bacterial, and MP infections, we explored whether these cell surface markers could aid in the differential diagnosis of these infections. Our findings not only confirm the utility of nCD64 and mCD169 in distinguishing between viral and common bacterial infections but also highlight their effectiveness in differentiating MP infections from both viral and bacterial etiologies. Furthermore, their diagnostic accuracy improves when combined with other commonly used biomarkers (Fig. 5). The combination of mCD169 ratio, IFN-α, and neutrophil and lymphocyte frequencies demonstrated strong diagnostic performance in identifying MP-infected children compared to those with viral or bacterial infections (Fig. S1).

To explore the potential reasons behind the observed differences in leukocyte activation patterns in children with MP infection, we further analyzed the relationship between these membrane surface markers and the patients’ nucleic acid and serological findings. The results revealed that both nCD64 and mCD169 were significantly reduced in DNA^-^IgM^+^ patients compared to DNA^+^IgM^+^ patients. Additionally, mCD169 levels were markedly lower in DNA^-^IgM^+^ patients than in DNA^+^IgM^-^ patients (Fig. 6). These findings suggest that the expression levels of activation markers are closely tied to the progression of the disease. The phases DNA^+^IgM^−^, IgM^+^DNA^+^, and IgM^+^DNA^−^ likely correspond to the early, progressive, and regressive stages of MP infection, respectively (5, 30). During the early and progressive phases, pathogens stimulate the expression of CD64 and CD169 either directly or indirectly. However, as the infection resolves and antibodies are produced, there is a downregulation of the immune response as the pathogens are cleared.

This study is not without its limitations. First, it was conducted at a single center with a limited cohort of pediatric patients, all presenting to the emergency department. The substantial age variation among the participants may have contributed to variability in the observed responses (31). Although the study included a range of prevalent infections, the sample size was insufficient for definitive validation of the findings. Expanding the study to multiple centers in future research would enhance its generalizability and robustness. Second, little is known about the kinetics of leukocyte activation markers, including their timing of expression after infection and how levels evolve over time. This study categorized patients by nucleic acid and serological test results as proxies for disease stage, but these did not always align with clinical histories. Future research should prioritize longitudinal sampling to clarify the prognostic value of these biomarkers in pediatric respiratory infections and their role in guiding treatment timing. Another important limitation is the exclusion of patients with mixed infections or immunocompromised status. Since MP co-infection with other pathogens is common in children (1), further studies should address these groups and assess whether leukocyte activation markers in immunocompromised patients can mount a similarly rapid and robust response to MP.

### Conclusions

The leukocyte activation markers nCD64 and mCD169 offer significant advantages over conventional biomarkers (e.g., WBC, CRP, and PCT) in infection diagnosis (32, 33). nCD64 rises sharply within hours as an early bacterial infection signal, while mCD169 exhibits high specificity for viral infections (8). Both biomarkers respond rapidly to pathogens and are less affected by non-infectious factors such as trauma or surgery (34). Their combined use enables reliable bacterial-viral differentiation, especially in immunocompromised patients and neonates, where traditional markers lack specificity (32, 34). Notably, both markers decline rapidly within 48 hours after infection resolution, providing a dynamic tool for monitoring antibiotic efficacy and prognosis (35).

This study is the first to characterize the leukocyte activation markers nCD64 and mCD169 in pediatric MP infections and to compare their expression with viral and other bacterial infections. We found that MP infection upregulates both nCD64 and mCD169, suggesting a link between the dynamics and disease progression. These markers may support MP diagnosis alongside epidemiological and imaging findings, but their clinical utility requires confirmation in prospective studies.

## SUPPLEMENTAL MATERIAL

Table S1 spectrum.01095-25-s0001.docx Pathogen identification in the study cohort.

Table S2 spectrum.01095-25-s0002.docx Correlation analysis between clinical indices and nCD64 or mCD169 in MP-infected patients.

Figure S1 spectrum.01095-25-s0003.docx ROC curves for distinguishing MP infections from healthy controls.

Figure S2 spectrum.01095-25-s0004.docx ROC curves for distinguishing MP infections from viral infections.

ASM does not own the copyrights to Supplemental Material that may be linked to, or accessed through, an article. The authors have granted ASM a non-exclusive, world-wide license to publish the Supplemental Material files. Please contact the corresponding author directly for reuse.

## References

[1] Li ZJ, Zhang HY, Ren LL, Lu QB, Ren X, Zhang CH, Wang YF, Lin SH, Zhang XA, Li J, et al.. 2021. Etiological and epidemiological features of acute respiratory infections in China. Nat Commun 12:5026. doi:10.1038/s41467-021-25120-6 34408158 PMC8373954

[2] Kutty PK, Jain S, Taylor TH, Bramley AM, Diaz MH, Ampofo K, Arnold SR, Williams DJ, Edwards KM, McCullers JA, Pavia AT, Winchell JM, Schrag SJ, Hicks LA. 2019. Mycoplasma pneumoniae among children hospitalized with community-acquired Pneumonia. Clin Infect Dis 68:5–12. doi:10.1093/cid/ciy419 29788037 PMC6552676

[3] Michael CA, Dominey-Howes D, Labbate M. 2014. The antimicrobial resistance crisis: causes, consequences, and management. Front Public Health 2:145. doi:10.3389/fpubh.2014.00145 25279369 PMC4165128

[4] Fontela PS, O’Donnell S, Papenburg J. 2018. Can biomarkers improve the rational use of antibiotics? Current opinion in infectious diseases 31:347–352. doi:10.1097/QCO.0000000000000467 29794541

[5] Gao L, Sun Y. 2024. Laboratory diagnosis and treatment of Mycoplasma pneumoniae infection in children: a review. Ann Med 56:2386636. doi:10.1080/07853890.2024.2386636 39097794 PMC11299444

[6] Loens K, Ieven M. 2016. Mycoplasma pneumoniae: current knowledge on nucleic acid amplification techniques and serological diagnostics. Front Microbiol 7:448. doi:10.3389/fmicb.2016.00448 27064893 PMC4814781

[7] Nuutila J, Hohenthal U, Laitinen I, Kotilainen P, Rajamäki A, Nikoskelainen J, Lilius E-M. 2007. Simultaneous quantitative analysis of FcgammaRI (CD64) expression on neutrophils and monocytes: a new, improved way to detect infections. J Immunol Methods 328:189–200. doi:10.1016/j.jim.2007.09.002 17905303

[8] Bourgoin P, Soliveres T, Ahriz D, Arnoux I, Meisel C, Unterwalder N, Morange P-E, Michelet P, Malergue F, Markarian T. 2019. Clinical research assessment by flow cytometry of biomarkers for infectious stratification in an Emergency Department. Biomark Med 13:1373–1386. doi:10.2217/bmm-2019-0214 31617736

[9] Bedin A-S, Makinson A, Picot M-C, Mennechet F, Malergue F, Pisoni A, Nyiramigisha E, Montagnier L, Bollore K, Debiesse S, Morquin D, Veyrenche N, Renault C, Foulongne V, Bret C, Bourdin A, Le Moing V, Van de Perre P, Tuaillon E. 2021. Monocyte CD169 expression as a biomarker in the early diagnosis of coronavirus disease 2019. J Infect Dis 223:562–567. doi:10.1093/infdis/jiaa724 33206973 PMC7717347

[10] van de Ven NLM, Bongers SH, Spijkerman R, Koenderman L, Leenen LPH, Hietbrink F, COVPACH study group. 2023. Point-of-care neutrophil CD64 as a rule in diagnostic test for bacterial infections in the emergency department. BMC Emerg Med 23:28. doi:10.1186/s12873-023-00800-2 36915043 PMC10010956

[11] van der Poll T, van de Veerdonk FL, Scicluna BP, Netea MG. 2017. The immunopathology of sepsis and potential therapeutic targets. Nat Rev Immunol 17:407–420. doi:10.1038/nri.2017.36 28436424

[12] Asadullah K, Woiciechowsky C, Döcke WD, Egerer K, Kox WJ, Vogel S, Sterry W, Volk HD. 1995. Very low monocytic HLA-DR expression indicates high risk of infection--immunomonitoring for patients after neurosurgery and patients during high dose steroid therapy. Eur J Emerg Med 2:184–190. doi:10.1097/00063110-199512000-00003 9422205

[13] Esposito S, Argentiero A, Gramegna A, Principi N. 2021. Mycoplasma pneumoniae: a pathogen with unsolved therapeutic problems. Expert Opin Pharmacother 22:1193–1202. doi:10.1080/14656566.2021.1882420 33544008

[14] Kumar S, Kumar S. 2023. Mycoplasma pneumoniae: among the smallest bacterial pathogens with great clinical significance in children. Indian J Med Microbiol 46:100480. doi:10.1016/j.ijmmb.2023.100480 37741157

[15] Subspecialty Group of Respiratory tSoPCMA, China National Clinical Research Center of Respiratory D, Editorial Board CJoP. 2024. Evidence-based guideline for the diagnosis and treatment of Mycoplasma pneumoniae pneumonia in children (2023). Chinese J Pediat 62:1137–1144. doi:10.1002/ped4.12469 39563040

[16] Stuckrad SL von, Klotsche J, Biesen R, Lieber M, Thumfart J, Meisel C, Unterwalder N, Kallinich T. 2020. SIGLEC1 (CD169) is a sensitive biomarker for the deterioration of the clinical course in childhood systemic lupus erythematosus. Lupus (Los Angel) 29:1914–1925. doi:10.1177/0961203320965699 PMC7684796 33081587

[17] Sakumura N, Yokoyama T, Usami M, Hosono Y, Inoue N, Matsuda Y, Tasaki Y, Wada T. 2023. CD169 expression on monocytes as a marker for assessing type I interferon status in pediatric inflammatory diseases. Clin Immunol 250:109329. doi:10.1016/j.clim.2023.109329 37061149

[18] Gatti A, Fassini P, Mazzone A, Rusconi S, Brando B, Mistraletti G. 2023. Kinetics of CD169, HLA-DR, and CD64 expression as predictive biomarkers of SARS-CoV2 outcome. J Anesth Analg Crit Care 3:6. doi:10.1186/s44158-023-00090-x 37386613 PMC10041484

[19] Jukema BN, de Hond TAP, Kroon M, Maranus AE, Koenderman L, Kaasjager KAH. 2024. Point-of-care neutrophil and monocyte surface markers differentiate bacterial from viral infections at the emergency department within 30 min. J Leukoc Biol 115:714–722. doi:10.1093/jleuko/qiad163 38169315

[20] Domitien Payet L, Bedin AS, Desselas É, Marie-Jeanne C, Mollevi C, Malergue F, Bourgoin P, Van de Perre P, Tuaillon É, Jeziorski É. 2024. Leukocyte activation patterns in hospitalized children: comparing SARS-CoV-2, bacterial infections, and inflammatory pathologies. J Leukoc Biol 116:830–837. doi:10.1093/jleuko/qiae093 38648502

[21] Pratesi C, De Rosa R, Pivetta E, Vattamattathil K, Malipiero G, Fontana DE, Basaglia G, Doretto P. 2025. Validation of monocyte CD169 expression as a valuable rapid diagnostic marker of SARS-CoV-2 and other acute viral infections. Am J Clin Pathol 163:340–349. doi:10.1093/ajcp/aqae127 39305084

[22] García-Salido A, de Azagra-Garde AM, García-Teresa MA, Caro-Patón GDL, Iglesias-Bouzas M, Nieto-Moro M, Leoz-Gordillo I, Niño-Taravilla C, Sierra-Colomina M, Melen GJ, Ramírez-Orellana M, Serrano-González A. 2019. Accuracy of CD64 expression on neutrophils and monocytes in bacterial infection diagnosis at pediatric intensive care admission. Eur J Clin Microbiol Infect Dis 38:1079–1085. doi:10.1007/s10096-019-03497-z 30712229

[23] Bourgoin P, Soliveres T, Barbaresi A, Loundou A, Belkacem IA, Arnoux I, Bernot D, Loosveld M, Morange P-E, Michelet P, Malergue F, Markarian T. 2021. CD169 and CD64 could help differentiate bacterial from CoVID-19 or other viral infections in the emergency department. Cytometry A 99:435–445. doi:10.1002/cyto.a.24314 33491921 PMC8014466

[24] Lai J-F, Zindl CL, Duffy LB, Atkinson TP, Jung YW, van Rooijen N, Waites KB, Krause DC, Chaplin DD. 2010. Critical role of macrophages and their activation via MyD88-NFκB signaling in lung innate immunity to Mycoplasma pneumoniae. PLoS One 5:e14417. doi:10.1371/journal.pone.0014417 21203444 PMC3009709

[25] Kårehed K, Dimberg A, Dahl S, Nilsson K, Oberg F. 2007. IFN-gamma-induced upregulation of Fcgamma-receptor-I during activation of monocytic cells requires the PKR and NFkappaB pathways. Mol Immunol 44:615–624. doi:10.1016/j.molimm.2006.01.013 16516295

[26] Habbeddine M, Verthuy C, Rastoin O, Chasson L, Bebien M, Bajenoff M, Adriouch S, den Haan JMM, Penninger JM, Lawrence T. 2017. Receptor activator of NF-κB orchestrates activation of antiviral memory CD8 T cells in the spleen marginal zone. Cell Rep 21:2515–2527. doi:10.1016/j.celrep.2017.10.111 29186688 PMC5723674

[27] Bourgoin P, Biéchelé G, Ait Belkacem I, Morange P-E, Malergue F. 2020. Role of the interferons in CD64 and CD169 expressions in whole blood: relevance in the balance between viral- or bacterial-oriented immune responses. Immun Inflamm Dis 8:106–123. doi:10.1002/iid3.289 32031762 PMC7016842

[28] Liang SY, Theodoro DL, Schuur JD, Marschall J. 2014. Infection prevention in the emergency department. Ann Emerg Med 64:299–313. doi:10.1016/j.annemergmed.2014.02.024 24721718 PMC4143473

[29] Larsen FF, Petersen JA. 2017. Novel biomarkers for sepsis: a narrative review. Eur J Intern Med 45:46–50. doi:10.1016/j.ejim.2017.09.030 28965741

[30] Rowlands RS, Meyer Sauteur PM, Beeton ML, On Behalf Of The Escmid Study Group For M, Chlamydia Infections E. 2024. Mycoplasma pneumoniae: not a typical respiratory pathogen. J Med Microbiol 73:001910. doi:10.1099/jmm.0.001910 39475213 PMC11523975

[31] Yu W, Yu Y, Sun S, Lu C, Zhai J, Lei Y, Bai F, Wang R, Chen J. 2024. Immune alterations with aging: mechanisms and intervention strategies. Nutrients 16:3830. doi:10.3390/nu16223830 39599617 PMC11597283

[32] Venturini S, Crapis M, Zanus-Fortes A, Orso D, Cugini F, Fabro GD, Bramuzzo I, Callegari A, Pellis T, Sagnelli V, et al.. 2025. Can nCD64 and mCD169 biomarkers improve the diagnosis of viral and bacterial respiratory syndromes in the emergency department? A prospective cohort pilot study. Infection 53:679–691. doi:10.1007/s15010-024-02468-7 39821738

[33] Cong S, Ma T, Di X, Tian C, Zhao M, Wang K. 2021. Diagnostic value of neutrophil CD64, procalcitonin, and interleukin-6 in sepsis: a meta-analysis. BMC Infect Dis 21:384. doi:10.1186/s12879-021-06064-0 33902476 PMC8072745

[34] Bourgoin P, Lediagon G, Arnoux I, Bernot D, Morange P-E, Michelet P, Malergue F, Markarian T. 2020. Flow cytometry evaluation of infection-related biomarkers in febrile subjects in the emergency department. Future Microbiol 15:189–201. doi:10.2217/fmb-2019-0256 32065550

[35] Shiley KT, Lautenbach E, Lee I. 2010. The use of antimicrobial agents after diagnosis of viral respiratory tract infections in hospitalized adults: antibiotics or anxiolytics? Infect Control Hosp Epidemiol 31:1177–1183. doi:10.1086/656596 20923284 PMC3219040

