# Supplementary material for: Leukocyte activation patterns in children with Mycoplasma pneumoniae infection: a comparison with viral and bacterial infections
Source: Microbiol Spectr. 2025 Oct 29;13(12):e01095-25. doi: 10.1128/spectrum.01095-25 (PMC12671156; doi:10.1128/spectrum.01095-25)
Supplement: Table S1 — Pathogen identification in the study cohort. [file spectrum.01095-25-s0001.docx]

Supplemental Table 1. Pathogen Identification in the Study Cohort

| Pathogen identified | Number isolated (n= 20) |
| --- | --- |
| **Bacterial** |  |
| Streptococcus pneumoniae | 8 (40.0%) |
| Staphylococcus aureus | 5 (25.0%) |
| Haemophilus influenzae | 4 (20.0%) |
| Klebsiella pneumoniae | 1 (5.0%) |
| Catamorium spp | 1 (5.0%) |
| Legionella pneumophila | 1 (5.0%) |
|  |  |
| **Virus** | Number isolated (n=42) |
| Flu A virus | 9 (21.4%) |
| Flu B virus | 9 (21.4%) |
| Rhinovirus | 6 (14.3%) |
| Human Respiratory Syncytial Virus | 5 (11.9%) |
| Human metapneumovirus | 4 (9.5%) |
| Adenovirus | 3 (7.1%) |
| Coronavirus | 2 (4.8%) |
| EB virus | 2 (4.8%) |
| SARS-CoV-2 | 1 (2.4%) |
| Parainfluenza virus | 1 (2.4%) |

A total of 42 viral detections were identified among the 33 patients. Seven individuals exhibited dual viral infections, including two cases of rhinovirus combined with adenovirus, two cases of rhinovirus combined with variant pulmonary virus, one case of human respiratory syncytial virus combined with adenovirus, one case of variant pulmonary virus-combined with coronavirus, and one case of parainfluenza combined with coronavirus.
