# Supplementary material for: Leukocyte activation patterns in children with Mycoplasma pneumoniae infection: a comparison with viral and bacterial infections
Source: Microbiol Spectr. 2025 Oct 29;13(12):e01095-25. doi: 10.1128/spectrum.01095-25 (PMC12671156; doi:10.1128/spectrum.01095-25)
Supplement: Table S2 — Correlation analysis between clinical indices and nCD64 or mCD169 in MP-infected patients. [file spectrum.01095-25-s0002.docx]

Supplemental Table 2. The correlation analysis between clinical indices and nCD64 or mCD169 in MP-infected patients

|  | | CRP | LEU | Neu(%) | Mono(%) | Lym(%) | neutrophil | monocyte | lymphocyte | B(%) | T(%) | Th(%) | Tc(%) |
| --- | --- | --- | --- | --- | --- | --- | --- | --- | --- | --- | --- | --- | --- |
| CD64ratio | *r* | .589** | -0.002 | 0.125 | 0.162 | -0.158 | 0.047 | 0.116 | -0.110 | .472** | -.304* | -0.077 | -.268* |
|  | *P* | 0.000 | 0.989 | 0.326 | 0.201 | 0.213 | 0.711 | 0.360 | 0.385 | 0.000 | 0.014 | 0.546 | 0.032 |
| CD169ratio | *r* | 0.028 | -0.128 | 0.122 | 0.057 | -0.130 | -0.083 | -0.057 | -0.149 | .286^*^ | -0.151 | -0.073 | -0.107 |
|  | *P* | 0.824 | 0.312 | 0.335 | 0.657 | 0.307 | 0.514 | 0.652 | 0.241 | 0.022 | 0.233 | 0.564 | 0.399 |
| CD64index | *r* | .658^**^ | 0.014 | 0.131 | 0.149 | -0.170 | 0.068 | 0.130 | -0.118 | .438^**^ | -.280^*^ | -0.057 | -.274^*^ |
|  | *P* | 0.000 | 0.910 | 0.301 | 0.240 | 0.178 | 0.594 | 0.306 | 0.353 | 0.000 | 0.025 | 0.653 | 0.028 |
| CD169index | *r* | 0.016 | -0.153 | 0.189 | 0.063 | -0.205 | -0.077 | -0.074 | -0.224 | 0.181 | -0.089 | -0.043 | -0.063 |
|  | *P* | 0.903 | 0.228 | 0.136 | 0.620 | 0.103 | 0.545 | 0.559 | 0.076 | 0.152 | 0.483 | 0.734 | 0.618 |

Continued

|  | | Tc(%) | Tratio | NK(%) | AbsB | AbsT | AbsTh | AbsTc | AbsNK | HRP | IFNα | IFNγ | IL6 | IL8 | IL10 |
| --- | --- | --- | --- | --- | --- | --- | --- | --- | --- | --- | --- | --- | --- | --- | --- |
| CD64ratio | *r* | -.268^*^ | 0.060 | -0.145 | 0.182 | -0.207 | -0.151 | -0.225 | -0.162 | -0.030 | 0.113 | .412^**^ | .521^**^ | .536^**^ | 0.131 |
|  | *P* | 0.032 | 0.638 | 0.253 | 0.149 | 0.101 | 0.233 | 0.074 | 0.200 | 0.812 | 0.424 | 0.002 | 0.000 | 0.000 | 0.355 |
| CD169ratio | *r* | -0.107 | -0.017 | -0.134 | 0.094 | -0.216 | -0.170 | -0.239 | -0.165 | -0.077 | .535^**^ | .560^**^ | 0.011 | -0.105 | 0.273 |
|  | *P* | 0.399 | 0.895 | 0.291 | 0.460 | 0.087 | 0.180 | 0.058 | 0.192 | 0.547 | 0.000 | 0.000 | 0.940 | 0.460 | 0.050 |
| CD64index | *r* | -.274^*^ | 0.080 | -0.139 | 0.144 | -0.197 | -0.145 | -0.217 | -0.173 | -0.044 | 0.019 | .366^**^ | .529^**^ | .535^**^ | 0.015 |
|  | *P* | 0.028 | 0.527 | 0.275 | 0.257 | 0.118 | 0.253 | 0.085 | 0.172 | 0.730 | 0.894 | 0.007 | 0.000 | 0.000 | 0.918 |
| CD169index | *r* | -0.063 | -0.038 | -0.095 | -0.033 | -.258^*^ | -0.219 | -.264^*^ | -0.166 | -0.096 | .536^**^ | .470^**^ | -0.042 | -0.157 | .372^**^ |
|  | *P* | 0.618 | 0.763 | 0.454 | 0.796 | 0.040 | 0.082 | 0.035 | 0.189 | 0.453 | 0.000 | 0.000 | 0.765 | 0.268 | 0.007 |
