# Supplementary material for: Leukocyte activation patterns in children with Mycoplasma pneumoniae infection: a comparison with viral and bacterial infections
Source: Microbiol Spectr. 2025 Oct 29;13(12):e01095-25. doi: 10.1128/spectrum.01095-25 (PMC12671156; doi:10.1128/spectrum.01095-25)
Supplement: Figure S1 — ROC curves for distinguishing MP infections from healthy controls. [file spectrum.01095-25-s0003.docx]

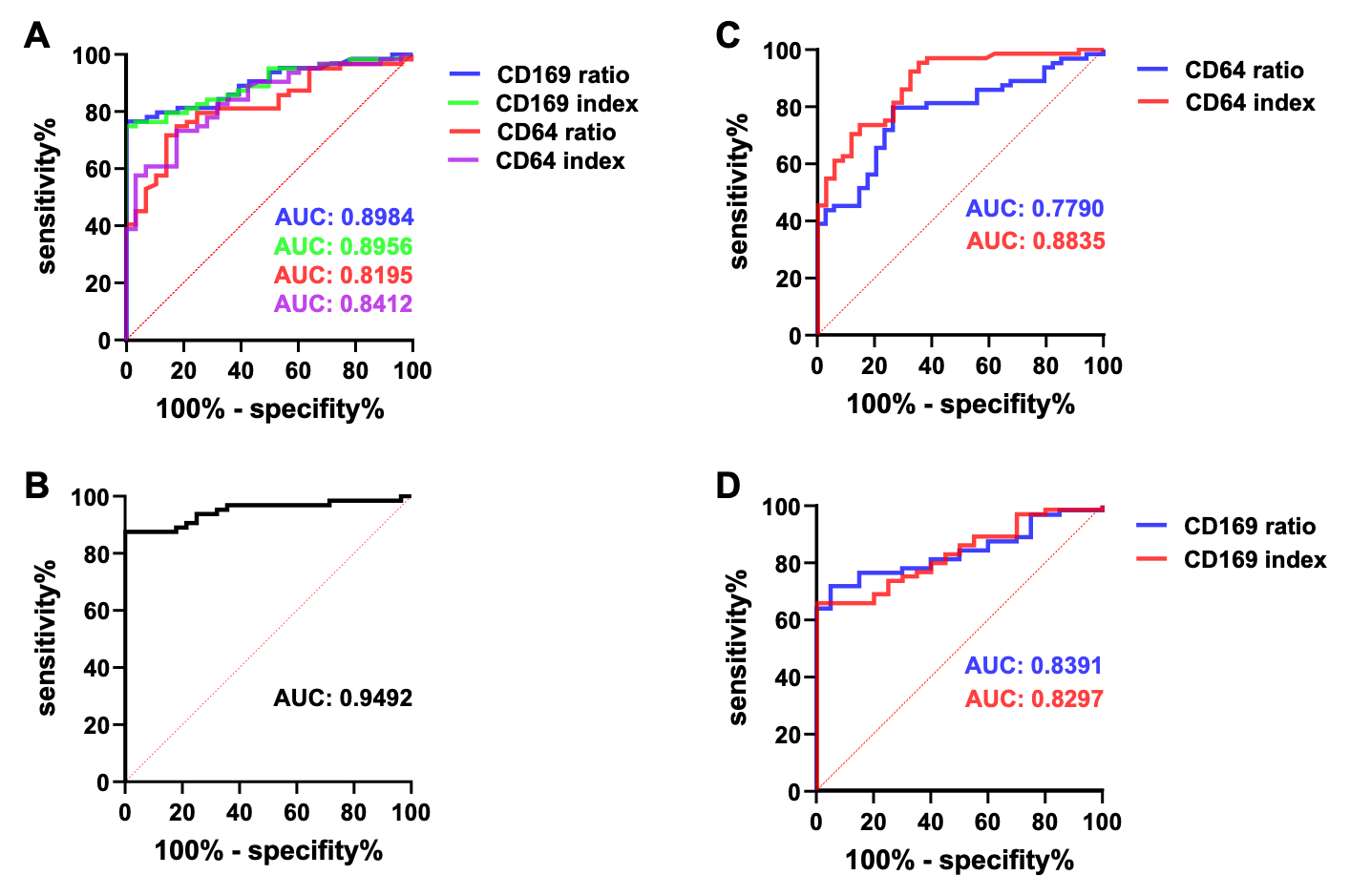


**Supplemental Figure 1.** (A) ROC curves for distinguishing MP infections from healthy controls using CD64 ratio, CD64 index, CD169 ratio and CD169 index. (B) ROC curve for MP infection detection in healthy controls using a combination of CD64 ratio, CD64 index and CD169 ratio. (C) ROC curves for differentiating MP infections from viral infections using CD64 ratio and CD64 index. (D) ROC curves for differentiating MP infections from bacterial infections using CD169 ratio and CD169 index. The area under the curve (AUC) values are provided for each analysis.
