# Supplementary material for: Leukocyte activation patterns in children with Mycoplasma pneumoniae infection: a comparison with viral and bacterial infections
Source: Microbiol Spectr. 2025 Oct 29;13(12):e01095-25. doi: 10.1128/spectrum.01095-25 (PMC12671156; doi:10.1128/spectrum.01095-25)
Supplement: Figure S2 — ROC curves for distinguishing MP infections from viral infections. [file spectrum.01095-25-s0004.docx]

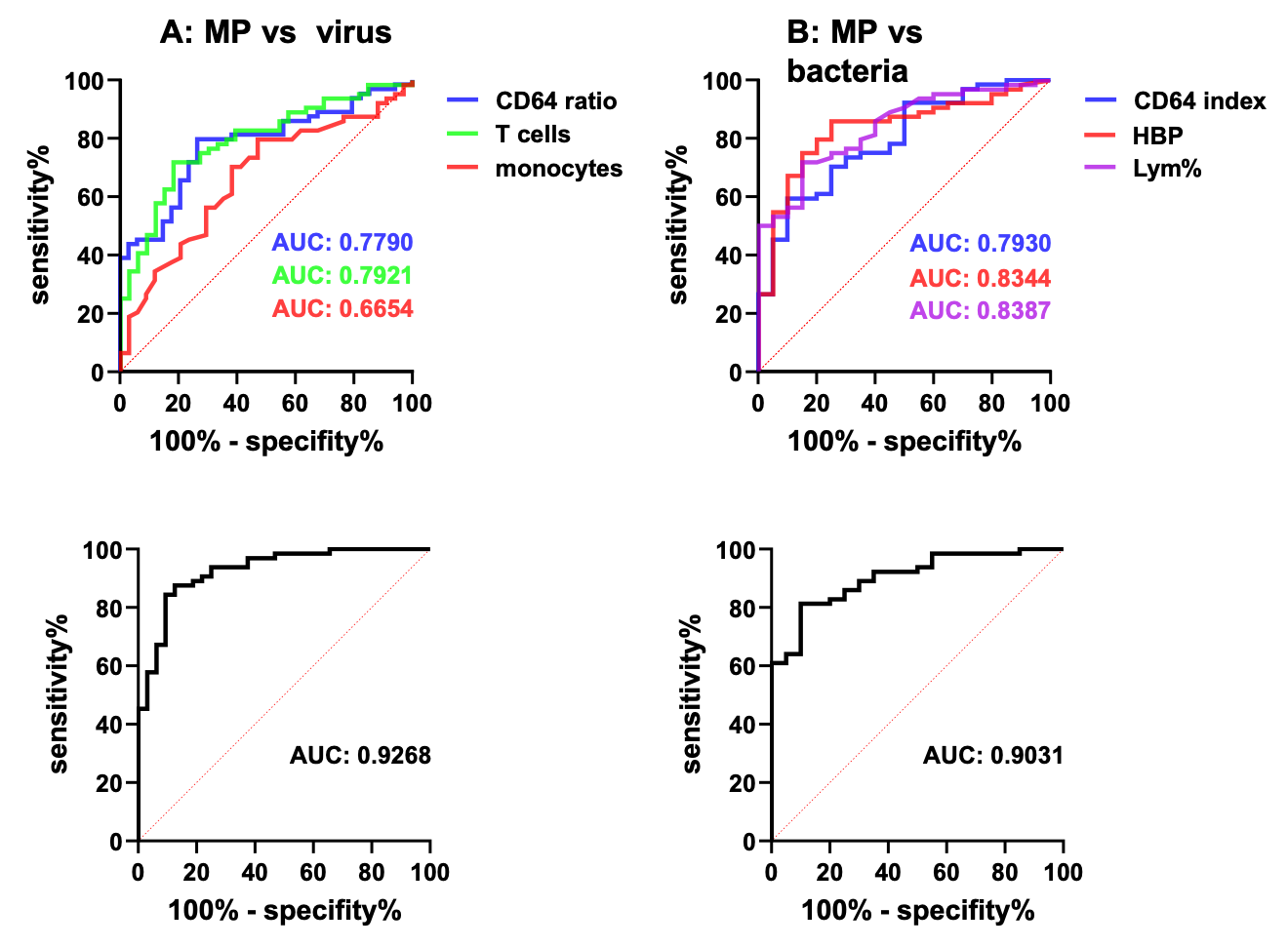


**Supplemental Figure 2.** (A) ROC curves for distinguishing MP infections from viral infections using individual markers, including the CD64 ratio, absolute T cell count, and monocytes count (upper panel), or their combination (lower panel). (B) ROC curves for distinguishing MP infections from bacterial infections using individual markers, including the CD64 index, heparin-binding protein (HBP) and lymphocyte frequency (Lym%) (upper panel), or their combination (lower panel).
